# The Impact of Sleep Disorders on Cardiovascular Health: Mechanisms and Interventions

**DOI:** 10.7759/cureus.49703

**Published:** 2023-11-30

**Authors:** Rushi Sarode, Prafulla P Nikam

**Affiliations:** 1 Medicine, Jawaharlal Nehru Medical College, Datta Meghe Institute of Higher Education and Research, Wardha, IND; 2 Anatomy, Jawaharlal Nehru Medical College, Datta Meghe Institute of Higher Education and Research, Wardha, IND

**Keywords:** cardiovascular health, cardiovascular risk, sleep timing, sleep disorder, sleep health

## Abstract

This comprehensive review article explores the intricate mechanisms at work and possible remedies for the connection between sleep issues and cardiovascular health. Sleep disorders, which include conditions like insomnia and sleep apnea, are drawing increasing amounts of attention due to their serious detrimental consequences on cardiovascular health. This article carefully examines the body of existing evidence to explain the intricate mechanisms that connect sleep disruptions to cardiovascular issues. Mechanisms include inflammation, disruption of the autonomic nervous system, endothelial dysfunction, and aberrant metabolic processes all have an impact on these pathways. The study also looks at a variety of existing and novel therapeutic modalities that aim to minimize the detrimental effects of sleep disruptions on cardiovascular health. This includes evaluating the effectiveness of lifestyle changes, pharmaceutical interventions, and behavioural therapy for enhancing sleep quality and hence preserving cardiovascular health. By synthesising and presenting the most recent study data, this article offers valuable insights into the complex relationships between sleep patterns, cardiovascular function, and potential therapeutics. These results provide a solid foundation for guiding future research endeavours and clinical judgements. Pharmacotherapy is a possibility for momentary relief. Cardiovascular illness has been linked to the sensorimotor problem known as restless legs syndrome (RLS), which causes a strong impulse to move the legs. Sleep disruption caused by RLS-related leg movements leads to sympathetic activation, elevated blood pressure, impaired vascular function, and potential iron deficiency. Treating the underlying iron deficiency, when present, and medications targeting dopamine receptors or regulating calcium channels are the primary interventions for RLS. In conclusion, sleep disorders significantly impact cardiovascular health through multiple mechanisms. Early detection, accurate diagnosis, and appropriate interventions are crucial for mitigating associated cardiovascular risks. Multidisciplinary approaches including lifestyle modifications, behavioral interventions, and targeted pharmacotherapy have shown promise in improving sleep quality and cardiovascular outcomes. Further research is needed to enhance our understanding of the complex interplay between sleep disorders and cardiovascular health, leading to the development of more effective interventions and improved patient outcomes.

## Introduction and background

For the human body as a whole, as well as for each organ and system, sleep is crucial for good health. Increased cardiovascular risks and degradation in human body functioning are linked to sleep disorders including insomnia, sleep-disordered breathing, fragmented sleep, and sleep deprivation [1]. Even while sleep plays a crucial part in preserving and enhancing physical and mental health, many people either don't get enough sleep or have sleeping disorders. This overview emphasizes the biological purposes of sleep, various sleep disorders, and lifestyle and sleep hygiene measures that can enhance sleep. Getting adequate sleep has positive effects on your hormone balance, immunity, reproductive health, mental health, cognition, memory consolidation, and cardiovascular health. Sleep disorders include insomnia, sleep apnea, and circadian rhythm disorders, as well as sleep disruption brought on by lifestyle choices, environmental factors, or other medical conditions, which can cause significant morbidity. Additionally, these diseases may exacerbate already present mental and physiological issues [2]. Increased energy expenditure is caused by changes to the circadian pattern of expression of a variety of metabolic genes in the liver, skeletal muscle, and adipose tissue [3]. A crucial component of both physical and mental health is getting enough sleep. Short sleep duration or poor sleep quality are major contributors to the widespread problem of sleep deficit in contemporary life. Some of the causes of inadequate sleep include occupation, social demands, psychological problems, physical disorders, and sleep disorders [4]. To sustain a healthy lifestyle, a synchronization between wakefulness and sleep must be well-balanced. An individual's natural sleep demand and circadian rhythm are the foundations of optimal sleep. The disruption of either one or both of these essential components may lead to daytime dysfunction, non-restorative sleep, and/or a diminished sense of well-being. Despite being more common in the general population, circadian rhythm sleep wake disorders (CRSWDs) are less well known in the medical community than other sleep disorders such as sleep apnea, insomnia, and narcolepsy. CRSWDs include irregular sleep-wake rhythm disorder, delayed sleep phase disorder, advanced sleep phase disorder, jet lag disorder, and non-24-hour sleep-wake disorder [5]. In addition to lowering quality of life, sleep difficulties have been linked to a range of medical problems that are bad for your health. Studies have linked sleep disturbances to greater rates of cardiovascular risk factors, higher rates of vascular outcomes, and higher rates of vascular mortality. However, most of them have concentrated on the dangers of breathing disorders while sleeping, while paying little attention to subjective sleep symptoms like sleep quality. Poor sleep quality has lately been linked to cognitive decline in elderly Japanese people. A nationwide US survey found no link between sleep hygiene and arterial hypertension [6]. Reduced sleep quantity and/or quality has also been linked to increased sympathetic nervous system activity, which has been linked to CVD risk factors like hypertension and diabetes [7]. After adjusting for socioeconomic and demographic risk variables and comorbidities, epidemiological studies have revealed that short sleep duration is linked to an increased prevalence of cardiovascular disorders, including coronary artery disease, hypertension, arrhythmias, diabetes, and obesity [8].

## Review

Search methodology

**Figure 1:**
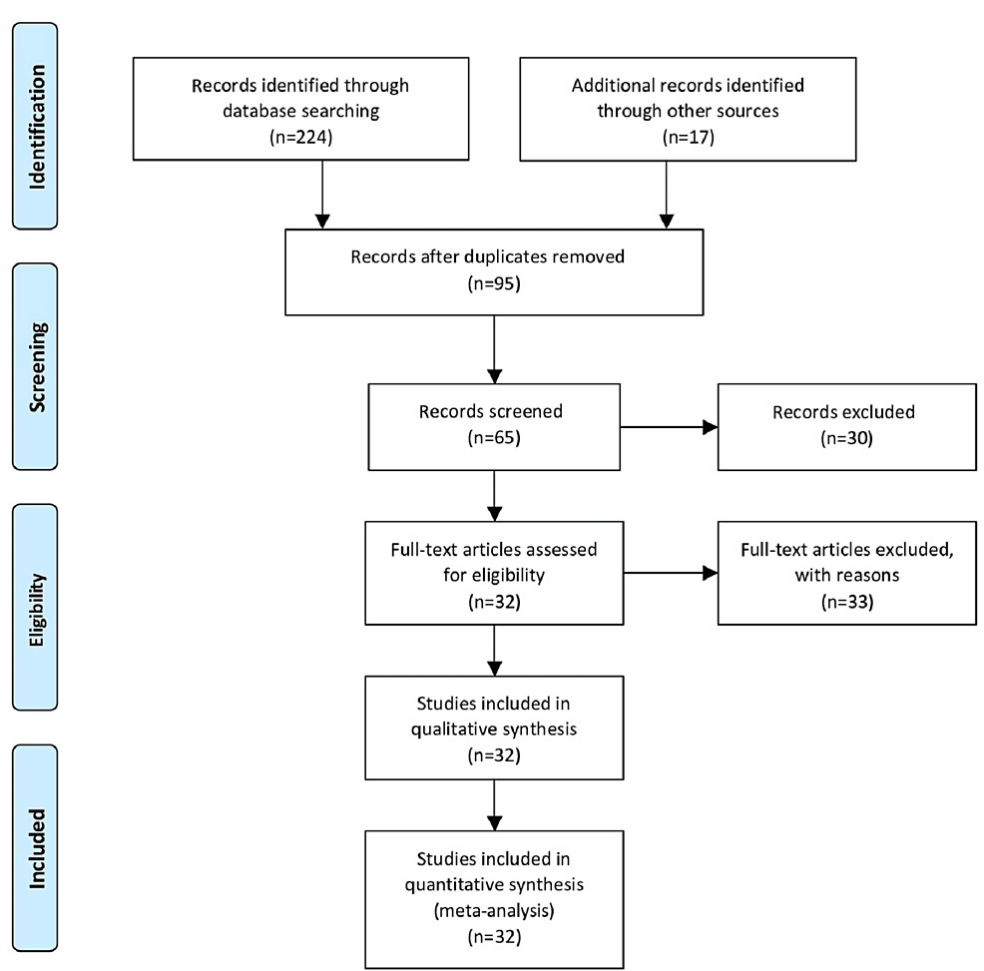
The PRISMA flowchart for literature search. PRISMA: Preferred Reporting Items for Systematic Reviews and Meta-Analyses

We searched MEDLINE via PubMed and CENTRAL DATABASE via the Cochrane Library. The search was done using the keywords: cardiovascular health, cardiovascular risk, sleep timing, sleep disorder, and sleep health. Furthermore, we screened the reference list of the potentially relevant studies to seek additional studies. Studies retrieved from these electronic searches and relevant references included in the bibliography of those studies were reviewed. Figure 1 shows the Preferred Reporting Items for Systematic Reviews and Meta-Analyses (PRISMA) flow diagram for the literature search.

Sleep disorders and their impacts on health

In the past, sleep disturbances were always considered to be a comorbidity of depression. Sleep issues were rarely treated because it was often believed that sleep disruptions would go away as an accompanying symptom of treating depression. There is a lot of recent evidence that suggests sleep problems come before depression [9]. Sleep disorders can disturb the normal functioning and structure of sleep, including obstructive sleep apnea (OSA), insomnia, and sleep deprivation. Understanding the mechanisms at play is essential for creating successful interventions because sleep disturbances can have a considerable influence on cardiovascular health. Numerous sleep disorders, including insomnia, restless leg syndrome, and sleep apnea, have been connected to cardiovascular issues. Self-reported sleep duration is frequently used in clinical practice and research because it is simple to use and may be applied to a large population. However, the inadequacies of self-reported sleep length were brought to light by the growing accessibility of modern technology that helps better assessments of sleep [10]. Sleep is a crucial component in promoting health. According to research undertaken over the past 10 years, sleep disturbance has a significant impact on the beginning and progression of many serious medical disorders, including cancer and cardiovascular disease, as well as the prevalence of depression. The field has increasingly focused on understanding the biological mechanisms underlying these effects. In addition to considering the dynamics of sleep disruption, sleep deprivation, and insomnia on antiviral immune responses with implications for vaccine responses and infectious disease risk and proinflammatory immune responses with implications for cardiovascular disease, cancer, and depression, this review emphasizes the role of sleep on adaptive and innate immunity [11]. Table 1 represents the sleep disorders and their impacts on health.

**Table 1:** Sleep disorders and their impacts on cardiovascular health.

Sleep Disorder	Impact on Cardiovascular Health
Sleep apnea	Increases the risk of hypertension. Raises the risk of stroke. Contributes to irregular heartbeat (arrhythmia). Promotes the development of heart failure.
Insomnia	Linked to higher blood pressure. Increases the risk of heart disease. Associated with a higher risk of heart attacks. May lead to an unhealthy heart rhythm.
Restless Leg Syndrome	Disrupts sleep patterns. Linked to increased risk of hypertension. Can lead to poor quality of sleep. May contribute to cardiovascular problems.
Narcolepsy	May cause irregular heartbeat. Increases the risk of heart disease. Can lead to sudden muscle weakness (cataplexy) Linked to a higher risk of obesity.
Circadian Rhythm Disorders	Disruption can lead to higher blood pressure. Increases the risk of metabolic disorders. Linked to higher rates of heart disease. Disrupted sleep patterns impact heart health.

Sympathetic overactivity

People with sleep disorders such as obstructive sleep apnea syndrome, periodic limb movements in sleep syndrome, insomnia, and narcolepsy-cataplexy are more likely to experience heart difficulties. All of these disorders cause the heart's sympathetic tone to be raised because of problems with autonomic nervous system control. This higher cardiovascular sympathetic tone is anticipated to have a major impact on the increased risk of cardiovascular disease. Numerous stressors, such as intermittent hypoxia, fragmented sleep, brief hypercapnia, increased respiratory effort, and decreased sleep duration, may set off the pathophysiological cascade that leads to sympathetic overactivity. In these numerous sleep disorders, some or all of these triggers are present. In this article, we outline the many pathways that, depending on the type of sleep, can lead to sympathetic overactivity [12]. It was found that regulatory coupling and neuronal connections among sympathetic nerve activity (SNA), apnea, and ventilation were responsible for the alarming nocturnal oscillations in arterial pressure and SNA. When awake, during normal breathing, and normoxia, patients with OSA showed elevated levels of SNA, which exacerbated hypertension and caused organ damage [13]. Overactive sympathetic nervous system and hypertension are frequently linked to OSA. Hypoxia's impact on arterial chemoreceptors is primarily responsible for these correlations. Uncertainty surrounds the role of arousal from sleep [14].

Inflammation

Many people's quality of life is substantially impacted by sleep disorders, however, this illness is still not widely understood. It is believed that your food has a big impact on how well you sleep. Numerous dietary supplements have been used in an effort to support healthy sleep. However, other elements have a role in the relationship between nutrition and sleep. Dietary patterns and each person's digestive and metabolic functions have a considerable impact on how well-balanced their diets are in terms of nutritional aspects [15]. The sleeping disease known as obstructive sleep apnea syndrome (OSAS) is characterized by recurrent apnea, ongoing hypoxia, oxygen desaturation, and hypercapnia. According to earlier research, intermittent hypoxia (IH) circumstances in OSAS patients caused neuron destruction, particularly in the hippocampus and cortex, which in turn caused cognitive impairment, an important and unusual consequence of OSAS patients. In OSAS patients, the recurrent episodes of airway collapse and blockage led to apnea and arousal during sleep, which in turn caused IH and excessive daytime sleepiness (EDS), and ultimately aided in the development of inflammation. Different types of cognitive impairment might also be triggered by IH-mediated inflammation. Numerous studies have revealed that in addition to continuous positive airway pressure (CPAP) therapy and surgery, anti-inflammatory drugs may be able to cure the neurocognitive impairment brought on by IH [16].

Endothelial dysfunction

It has become clear that nocturnal intermittent hypoxia is strongly correlated with oxidative/nitrosative stress, an increase in pro-inflammatory markers, an imbalance in NO production, and endothelial dysfunction. The contribution of body mass index (BMI) requires more explanation. Treatment for OSA with CPAP has been shown to improve vascular health and the pro-inflammatory environment [13]. A rise in soluble intercellular adhesion molecule-1 (sICAM-1) levels is a sign of endothelial damage caused by severe OSAS. A rise in NT-proBNP levels is a sign of changes in right ventricular shape and function, which are primarily seen in patients with increased thrombin-antithrombin complex (TAT) and endothelin-1 levels. Patients with moderate-to-severe OSAS appear to be unaffected by long-term CPAP therapy, which may assist in explaining why CPAP does not appear to reduce cardiovascular risk [17].

Metabolic dysregulation

For healthy metabolism, sleep and circadian rhythms moderate or regulate daily physiological patterns. Metabolic dysregulation may result from sleep deficits brought on by irregular sleep patterns, insomnia with short sleep duration, sleep apnea, narcolepsy, circadian misalignment, shift work, night eating syndrome, and sleep-related eating disorders [18]. Alzheimer's disease and Parkinson's disease are two pathologic conditions that have been linked to dysfunction in melatonin release or production. Reduced melatonin has also been linked to several malignancies as well as mental, cardiovascular, genitourinary, and dermatological conditions such as atopic dermatitis, depression, myocardial infarction, vasculitides, and erectile dysfunction. Numerous medical disorders affecting various organ systems have been studied about the possible therapeutic benefits of melatonin [19]. When the endogenous biological clock and extrinsic light-dark cycle are out of synchrony, resynchronization with the external clock and environment is the goal of treatment. There may be severe suffering and dysfunction as a result. An overview of circadian neurobiology and the endogenous molecular clock is given in the opening paragraphs of this article, which also provides a brief historical context for the advancement of our understanding of circadian rhythms [20]. An underlying mechanism linking objective and subjective measures of sleep quality in older persons with insomnia may be dysregulation of autonomic activity, which may also have a negative impact on health [21].

Interventions for mitigating the impact of sleep disorders on cardiovascular health

Continuous Positive Airway Pressure (CPAP)

The most significant cause of illness and mortality in preterm newborns is respiratory distress, most specifically respiratory distress syndrome (RDS). Intermittent positive pressure ventilation (IPPV) with surfactant has been the standard treatment for newborns with increasing respiratory insufficiency, although it is intrusive and may cause lung and airway damage [22]. Chronic and becoming more common is OSA. OSAS has been linked to decreased quality of life, workplace mishaps, and auto accidents brought on by excessive daytime sleepiness in addition to accompanying cardiovascular comorbidities. Although continuous positive airway pressure is the gold standard for treating sleep apnea, it is unclear how it affects quality of life [23]. The foundation of customized therapy is the phenotyping of the pathophysiology of OSA, one of the most contentious issues in sleep medicine research. The potential for understanding the variation of endophytic qualities in patients with OSA, which accounts for the heterogeneity in the clinical presentation of the disease and, consequently, in the outcome of treatment, has been expanded by more sophisticated approaches [24]. OSA is a partial or total obstruction of the upper airway that alters the regular alveolar ventilation and sleep cycle. Snoring, mouth breathing, breathing pauses, restlessness, enuresis, and perspiration are examples of nocturnal symptoms. Sleepiness, which can be difficult to obtain from smaller children while taking a medical history, and poor academic performance in school-aged children are daytime repercussions. Up to 30% of children with OSA also have an attention deficit hyperactivity disorder diagnosis, which is a distinctive symptom present in pediatric patients but not adults [25].

Lifestyle

Over the past three decades, depression seems to have become more common. Even if this could be a byproduct of diagnostic procedures, modernity is probably playing a role in this development. There is now strong proof that several lifestyle factors contribute to depression's aetiology. While many of these characteristics can be changed, little attention is paid to them in the current management of depression, which still relies mostly on medicine and psychological therapy [26]. Weight loss, increased aerobic capacity, and altered sleep architecture were all achieved with the use of supervised aerobic exercise and a healthy diet [27].

Sleep Health

Extreme sleep duration's effects on cardiovascular outcomes and mortality risk are still debatable. We set out to measure the dose-response connections between sleep duration and risk of overall cardiovascular disease, coronary heart disease, and stroke [28].

Medicine

Melatonin is the main hormone in charge of controlling the sleep-wake cycle. Because it is easy to synthesize and may be administered orally, there is interest in employing it as a sleep aid. Additionally, it has been proposed that melatonin deficiency is at least partially to blame for sleep disorders because the hormone's production declines with ageing and is inversely correlated with the frequency of poor sleep quality [29].

Behavioral Therapies

As a result of its effectiveness, cognitive behavioural therapy for insomnia (CBT-I) is currently regarded as the first-line treatment for both simple insomnia and insomnia that co-occurs with other chronic diseases. The goals of this study are to give a thorough summary of the efficacy data (such as efficacy overall, by clinical and demographic factors, and by CBT-I formulation), as well as to talk about the future of CBT-I [30]. The results of this study show that while positive airway pressure (PAP) alone is inferior to CBT-I in terms of insomnia outcomes, PAP adherence is not significantly improved [31]. A major public health priority is the prevention of major depressive disorder (MDD). Effective preventive care could be directed at those with a high risk of acquiring MDD using strategies. Insomnia is a common precursor to incident and relapse events and is a reliable sign of depression. Insomnia can therefore be used as a beginning point to treat MDD [32]. Table 2 summarises the studies included in the review.

**Table 2:** Summary of the studies included in the review

Author	Year	Findings
Fang et al. [9].	2019	Sleep problems come before depression.
Antza et al. [10].	2021	Sleep has a major role in cardiovascular health.
Irwin [11].	2015	Role of sleep in immunity.
Tamisier et al. [12].	2018	Role of sympathetic activity in sleep disorders patient.
Abboud et al. [13].	2014	Relation between sympathetic nerve activity and sleep disorders.
Ferreira et al. [14].	2020	Relation between sympathetic activity and obstructive sleep apnea.
Zhao et al. [15].	2020	Effect of nutrition on sleep.
Liu et al. [16].	2020	Relation between obstructive sleep apnea and inflammation.
Harańczyk et al. [17].	2022	Endothelial dysfunction in obstructive sleep apnea patients.
Depner et al. [18].	2014	Metabolic dysregulation can result in sleep deficiency.
Vasey et al. [19].	2021	Dysfunction in melatonin causes sleep disorders.
Steele et al. [20].	2021	Circadian rhythm
Grimaldi et al. [21].	2020	Sleep insomnia may be due to dysregulation of autonomic activity.
Ho et al. [22].	2020	Continuous positive airway pressure (CPAP) has been used to prevent apnoea.
Batool-Anwar et al. [23].	2016	Obstructive sleep apnea is a chronic disorder associated with cardiovascular disorders.
Bosi et al. [24].	2021	Understanding of the variability of endotypic traits in Obstructive sleep apnea patients.
Wang et al. [25].	2021	Symptoms of obstructive sleep apnea. Attention deficit hyperactivity disorder is associated with obstructive sleep apnea in children.
Sarris et al. [26].	2014	Several lifestyle factors contribute to depression.
Roche et al. [27].	2018	Impact of long-term physical exercise and a balanced diet on sleep duration.
Yin et al. [28].	2017	Effect of sleep duration on cardiovascular health.
Poza et al. [29].	2022	Melatonin is the main hormone responsible for the sleep-wake cycle.
Muench et al. [30].	2022	Cognitive behavioral therapy for insomnia (CBT-I) is considered as first-line treatment for insomnia.
Ong et al. [31].	2020	Combining CBT-I with PAP is superior to positive airway pressure (PAP) alone for insomnia.
Drake et al. [32].	2022	Insomnia is a precursor to depression.

## Conclusions

In conclusion, the relationship between sleep disorders and cardiovascular health is a critical area of research that has provided valuable insights into how our sleep patterns affect our heart health. Over the years, numerous studies have revealed that sleep disorders, such as sleep apnea and insomnia, can have far-reaching consequences on our cardiovascular system. The mechanisms connecting these two seemingly unrelated aspects of health are complex yet fascinating. Sleep disorders can trigger a chain reaction of physiological changes, including increased sympathetic nervous system activity and inflammation. These changes can pave the way for more serious issues, like endothelial dysfunction, metabolic disturbances, and irregular blood pressure. Thankfully, researchers and healthcare professionals haven't been idle in the face of these challenges. They have been hard at work developing interventions to mitigate the negative impact of sleep disorders on cardiovascular health. Promising strategies include the use of CPAP devices for sleep apnea, adopting healthy lifestyle practices encompassing diet, exercise, and stress management, improving sleep hygiene, and even exploring medication and behavioural therapies to aid sleep quality. However, it is important to acknowledge that this is a field that continues to evolve. Each individual's response to interventions may differ, and there is always room for ongoing research and discovery. As we move forward, a collaborative approach between individuals and healthcare providers will remain crucial. By focusing on both improving sleep quality and maintaining cardiovascular wellness, we can strive for a healthier and more balanced life. To stay current and well-informed, it is advisable to consult the latest research articles and expert opinions from reputable scientific sources. In essence, our journey toward better sleep and heart health is an ongoing endeavour that requires dedication, cooperation, and a keen understanding of the intricate interplay between our rest and our heart.
